# Impact of 4th of July Fireworks on Spatiotemporal PM_2.5_ Concentrations in California Based on the PurpleAir Sensor Network: Implications for Policy and Environmental Justice

**DOI:** 10.3390/ijerph18115735

**Published:** 2021-05-27

**Authors:** Amirhosein Mousavi, Yiting Yuan, Shahir Masri, Greg Barta, Jun Wu

**Affiliations:** 1 Program in Public Health, Department of Environmental and Occupational Health, Susan and Henry Samueli College of Health Sciences, University of California, Irvine, CA 92697, USA; amirhom@hs.uci.edu (A.M.); yitingyu@usc.edu (Y.Y.); masris@hs.uci.edu (S.M.); 2 SciVfx Initiative, London, UK; tracingrays@gmail.com

**Keywords:** air quality, firework, particulate matter, low-cost sensors, PurpleAir, Independence Day

## Abstract

Fireworks are often used in celebration, causing short term, extremely high particulate matter air pollution. In recent years, the rapid development and expansion of low-cost air quality sensors by companies such as PurpleAir has enabled an understanding of air pollution at a much higher spatiotemporal resolution compared to traditional monitoring networks. In this study, real-time PM_2.5_ measurements from 751 PurpleAir sensors operating from June to July in 2019 and 2020 were used to examine the impact of 4th of July fireworks on hourly and daily PM_2.5_ concentrations at the census tract and county levels in California. American Community Survey (ACS) and CalEnviroScreen 3.0 data were used to identify correlations between PM_2.5_ measurements and socioeconomic status (SES). A two-step method was implemented to assure the quality of raw PM_2.5_ sensor data and sensor calibration against co-located reference instruments. The results showed that over 67% and 81% of counties experienced immediate impacts related to fireworks in 2019 and 2020, respectively. Relative to 2019, the peak PM_2.5_ concentrations on July 4th and 5th 2020 were, on average, over 50% higher in California, likely due to the COVID-19-related increase in the use of household-level fireworks. This increase was most pronounced in southern counties, which tend to have less strict firework-related regulations and a greater use of illegal fireworks. Los Angeles County experienced the highest July 4th daily PM_2.5_ levels both in 2019 (29.9 µg·m^−3^) and 2020 (42.6 µg·m^−3^). Spatial hot spot analyses generally showed these southern counties (e.g., Los Angeles County) to be regional air pollution hotspots, whereas the opposite pattern was seen in the north (e.g., San Francisco). The results also showed PM_2.5_ peaks that were over two-times higher among communities with lower SES, higher minority group populations, and higher asthma rates. Our findings highlight the important role that policy and enforcement can play in reducing firework-related air pollution and protecting public health, as exemplified by southern California, where policy was more relaxed and air pollution was higher (especially in 2020 when the 4th of July coincided with the COVID-19-lockdown period), and in disadvantaged communities where disparities were greatest.

## 1. Introduction

Extensive evidence has documented higher concentrations of fine particulate matter less than 2.5 micrometers in aerodynamic diameter (PM_2.5_) and co-pollutants (i.e., trace metals and water-soluble ions) related to firework displays on celebratory occasions [1,2,3,4]. During (and sometimes several days prior to or after) festivals and national holidays, daily PM_2.5_ concentrations have been shown to be 2 to 10 times greater than background levels [5,6,7]. Measurements have also shown elevated concentrations of trace metals, water-soluble ions, and polycyclic aromatic hydrocarbons (PAHs) in the atmosphere during firework episodes [8,9]. Findings from toxicological studies demonstrate that particles generated from fireworks can produce adverse effects in mammalian cells and lungs, underscoring the need to expand our understanding of the contribution of firework-related emissions to ambient air pollution and the implications to public health [3,10,11,12]. In the United States, the New Year and 4th of July holidays constitute major annual firework episodes that impact regional and local air quality, leading to short-term PM_2.5_ and gas-phase air pollution exposure [1,6].

To date, limited studies have characterized the PM_2.5_ mass and composition related to firework emissions during the 4th of July at a large scale in the U.S. Seidel et al. (2015) examined hourly and daily PM_2.5_ measurements using 315 Environmental Protection Agency (EPA)-operated air quality monitoring stations within the U.S. from 1999 to 2013 and found that PM_2.5_ concentrations were elevated on both July 4th and 5th and that 24 h PM_2.5_ levels were elevated by 42% on average [6]. Dickerson et al. (2017) further examined chemical species from filter-based PM_2.5_ samples collected from EPA-based monitoring stations from 2000 to 2014 in the U.S. and found statistically significant increases in the concentrations of firework-related species, such as barium, chlorine, copper, magnesium, potassium, and strontium, on July 4th, which persisted through July 5th [1]. However, a major drawback of large-scale studies using sparse monitoring stations is their inability to identify localized impacts and firework-related air pollution hotspots at a fine spatial resolution. 

In general, most of the studies to date that examined the impact of short-term firework displays on outdoor ambient PM_2.5_ used a similar approach by sampling the air across a limited number of fixed monitoring sites located on building rooftops or other remote monitoring sites [13,14], which are typically not within populated urban locations that would better represent large population exposures. In addition to the limited spatial resolution of measurements, the filter-based PM_2.5_ speciation measured by the U.S. EPA is often conducted every third or sixth day and therefore often misses, or only partially covers, key air pollution events (e.g., reporting data on July 3rd and 5th, but not July 4th), in turn failing to fully reflect the exposure at a high spatial and temporal resolution. Overall, previous studies that have shown firework episodes to increase regional PM_2.5_ concentrations are constrained to large spatial and temporal scales [10,15,16,17].

Recently, remote sensing and data from low-cost sensor networks have been used to capture short-term variations in PM_2.5_ concentrations at finer spatial resolutions, compared to routine and sometimes unavailable government-operated monitoring stations, during major air pollution events [18], such as wildfires [19,20]. Satellite aerosol optical depth combined with ground-based PM_2.5_ data have been used to measure the impact of fireworks on PM_2.5_ concentrations [21]. These studies, however, only reported elevated PM_2.5_ levels during major national firework ceremonies at the city and national level [12,18]. Thus, there is a need in exposure assessment for high spatiotemporal resolution analyses to better characterize the individual- and local-level exposure to firework-related PM_2.5_.

In the U.S., and globally, there has been a growing interest in the use of the widely distributed, densely located, and low-cost PurpleAir sensor network to characterize spatiotemporal variations in PM_2.5_ air pollution across communities and to understand both long-term and short-term exposure change resulting from wildfires and other major air pollution events [22,23,24,25]. PurpleAir sensors are usually deployed in households and within populated areas. This sensor network has the advantage of measuring PM pollution continuously over time and at a much higher spatial resolution relative to the EPA’s routine monitoring network, thus allowing for an improved characterization of local PM_2.5_ exposure during the 4th of July episode and other celebratory days in the year. However, concerns exist regarding the uncertainty and malfunctions (i.e., wireless communications loss, power outages, or other measurement interferences) of the low-cost PurpleAir sensor data as well as potential biases due to environmental conditions, which requires a systematic post-process approach to ensure sensor data quality, especially in long term studies [26,27,28]. While most of the previous studies suggest calibrating the PurpleAir sensor data to the nearest reference monitor with a correction factor as a result of multi-variate regression, accounting for environmental (e.g., relative humidity and temperature) and operational factors (i.e., sensor uptime after deployment) [23,29], a recent study in Los Angeles County [30] has developed a systematic multi-step quality control scheme using the readings from both of the sensor’s channels to minimize outliers and eliminate readings from malfunctioning sensors.

In this study, we examined time-resolved PM_2.5_ data from the PurpleAir sensor network during both control days (preceding and succeeding the 4th of July firework episode) and non-control days (during the 4th and 5th of July, or immediately after) in the state of California, U.S., to identify temporal trends and spatial hotspots of PM_2.5_ pollution related to firework emissions at the county and census tract level. We also assessed the effect of inter-county and inter-city pollution-related regulations and restrictions to help inform future policy.

## 2. Data and Methods

### 2.1. PurpleAir Sensor Network PM_2.5_ Data

The PurpleAir monitoring network consists of low-cost air pollution monitoring devices that started to be deployed in the U.S. and worldwide in 2017. The latest model (PA-II-SD) contains two PMS5003 sensors (Plantower, Beijing, China), which estimate particle mass concentrations based on light scattering [28]. Overall, PurpleAir PA-II sensors show excellent accuracy compared to reference PM_2.5_ measurements (i.e., R2 ~ 0.93–0.97) over a concentration range of 0 to 250 µg·m^−3^ [31]. Further details regarding the lab evaluation of PurpleAir sensors conducted by the South Coast Air Quality Management District (AQ-SPEC team) and other research groups can be found elsewhere [32,33]. Recent studies have suggested the use of the PurpleAir network to supplement regulatory monitors for PM_2.5_ exposure assessment [23]. In this study, 10 min PM_2.5_ concentration data during June and July in 2019 and 2020 were retrieved from the PurpleAir network using ThingSpeak’s API provided by the PurpleAir company [34]. A continued expansion of the PurpleAir network in California has resulted in an increase in the number of operating sensors from 231 in July 2017 to 751 in July 2019, and a further increase to 2095 in July 2020. In order to allow for a comparison between the years, this study only retrieved PM_2.5_ raw data from the individual outdoor devices that were in operation in both 2019 and 2020, amounting to 751 PurpleAir devices across the state. These sensors were primarily located in densely populated areas (Figure S1).

### 2.2. Post-Process of PurpleAir PM_2.5_ Data

Given the importance of sensor data calibration and quality control, we used a two-step approach to post-process the hourly PurpleAir PM_2.5_ data in order to minimize the impact of sensor malfunction, intra-sensor bias, and environmental and operational disruptions. This approach involved a systematic quality control and a calibration of the sensor data with reference monitors.

### 2.3. Systematic Quality Control

A comprehensive description of the quality control method used in this study can be found in a study by Lu et al. (2021) [30]. In summary, the systematic quality control consisted of the following five steps: (1) removing malfunctioning sensor data based on low frequencies of change (e.g., a 5-h moving standard deviation of zero) in their readings across time; (2) discarding apparent PM_2.5_ outliers with extreme hourly values (>500 μg·m^−3^) exceeding the sensor’s effective measurement range in both channels, or readings higher than three times the calculated median absolute deviation by one channel within a calendar month in our study period (i.e., June and July); (3) identifying periods of prolonged data interruption due to power outages or data communication losses using a 75% completeness criterion (four or more 10-minute measurements per hour for 18 h or more in a day); (4) evaluating the degree of agreement in dual-channel readings for each sensor within a given month of operation, based on calculated statistical anomality detection indicators according to the coefficient of determination R2 > 0.8 and a mean absolute error < 4 (given the reported low performance (high saturation) of PurpleAir sensors at high particle concentrations (>50 µg·m^−3^), leading to a higher measurement bias and uncertainty in the mean absolute error indicator, a mean absolute percentage error of >0.3 was adopted as an additional criterion to handle the sensor’s measurement limit at high particle concentrations); and (5) applying a linear regression to hourly readings for each sensor that had a neighboring sensor within 3 km (only applicable to sensors that reported data from just one channel). As part of this last step, the data from sensors that had an R2 < 0.6 or did not have a neighboring sensor within 3 km was discarded from the analysis. 

Following these steps, 0.8%, 2%, 6.6%, 1.7%, and 0.9% of the initial 1,081,440 hourly averaged PM_2.5_ measurements from 751 sensors across California during June and July in both 2019 and 2020 were discarded after the first, second, third, fourth, and fifth steps, respectively, resulting in 12.1% of the measurements being discarded in total. Overall, a regression of the U.S. EPA’s co-located air quality system (AQS) data against the original sensor data (within a 500-meter radius) had an R2 of 0.56 and a slope of 0.67. Spikes were also detected in the original sensor data compared to the AQS data, with a high bias of 2.5 µg·m^−3^. The quality-controlled data (*n* = 951,667) had a more robust dual-channel agreement with the co-located AQS data (R2: 0.86; slope: 0.92). These results agree with research conducted by Bi et al. (2021) that showed a stronger statistical agreement with the AQS data based on the quality-controlled sensor data [23].

### 2.4. Sensor Data Calibration

After the systematic quality control of the data, we used a spatially varying calibration method developed by Bi et al. (2020) for outdoor sensor data calibration. Greater details on this calibration method can be found elsewhere [23]. In short, using the data from paired outdoor low-cost PurpleAir sensors and AQS regulatory stations (54 outdoor sensors and 26 co-located AQS stations within a 500-m radius), a geographically weighted regression (GWR) was used to calibrate the sensors’ PM_2.5_ data, using temperature and relative humidity (RH) measurements from individual sensor recordings as covariates [23]. In addition, to account for the quality degradation over time [23,26,27,28,30,33], the total operating time of a sensor (the duration between the measurement time and the installation time) was used to adjust for the effect of sensor aging [23]. Lastly, the sensor uptime (the time during which a sensor is in consecutive operation from the last boot time) was used to adjust for the potential impact of sensor operational stability on data quality. The following linear GWR was used to describe the relationship between the bias of the PurpleAir measurements and four covariates [23]:

AQS PM_2.5i_ = β_0_(ui,vi) + β_1_(ui,vi). PurpleAir PM2.5_i_ + β_2_(ui,vi).T_i_ + β_3_(ui,vi).RH_i_ + β_3_(ui,vi).optime_i_ + β_4_(ui,vi).uptime_i_ + ϵ_i_ (1)

where β(ui,vi) indicates the vector of the location-specific parameter estimates, and (ui,vi) represents the geographic coordinates of location i. AQS PM_2.5_i and PurpleAir PM_2.5_i are the paired hourly PM_2.5_ measurements at location i. Ti, RHi, optimei, and uptimei represent temperature, relative humidity, operating time, and uptime of the PurpleAir sensor at location i, respectively. The optimal hyperparameters of the GWR (i.e., the kernel and bandwidth) were chosen based on the corrected Akaike information criterion. In this analysis, the optimal kernel was a Gaussian kernel, and the optimal bandwidth was 5401 nearest-neighboring points. All of the covariates were statistically significant at an α level of 0.05 in the GWR calibration model. The GWR was fitted using the R package “GW model” version 2.0. Finally, we compared the mean absolute differences of hourly measurements between 54 outdoor sensors and 26 AQS stations in both 2019 and 2020, based on the PM_2.5_ measurement ranges after and before calibration. 

### 2.5. Demographic and Socioeconomic Factors and the Urban vs. Rural Variable

The demographic and socioeconomic variables were derived from CalEnviroScreen (CES) 3.0 (OEHHA, Sacramento, CA, USA), which was developed by the California EPA to address the issue of environmental justice and identify disadvantaged communities (DACs) in the state. The CES tool examines multiple individual factors, including pollution burden, population demographics (e.g., total population, age, race/ethnicity), socioeconomic status (SES), and disease indicators, and integrates these into a composite overall CES score (0–100). A composite population characteristics score (0.1–10) is also derived for each census tract using the average percentiles for three disease indicators (e.g., asthma, cardiovascular disease, low birth weight) and five SES indicators (educational attainment, housing burden for low-income households, linguistic isolation, poverty, and unemployment). Details on this tool and a description of these variables can be found elsewhere [35]. In our analysis, we included total population, race/ethnicity, percent of elderly (>65 years of age) and children (<10 years of age), educational attainment, poverty, asthma rate, and the overall CES score at the census tract level. DACs, defined as the top 25% CES scoring census tracts, were also considered through the use of a binary variable [35]. Further, we classified census tracts as urban or rural areas based on rural–urban commuting area codes that were developed using measures of population density, urbanization, and daily commuting [36].

### 2.6. Spatial and Temporal Analysis

The hourly average, daily average, and weekly average PM_2.5_ concentrations were computed by averaging 10 min data using a 75% completeness criterion (≥4 10-minute measures per hour and ≥18 h in a day). These data were grouped by census tract and county in order to investigate the local and regional impacts. July 4th-related firework activity usually takes place gradually, beginning in the first few days of July and peaking on the 4th and 5th of July [30]. To quantify the short-term impact of firework emissions, normalized hourly average PM_2.5_ concentrations were investigated at the county level in 2019 and 2020 over three 24 h time periods before (7:00 p.m. July 3rd–6:59 p.m., July 4th), during (7:00 p.m. July 4th–6:59 p.m. July 5th), and after (7:00 p.m. July 5th–6:59 p.m. July 6th) the main 4th of July firework displays.

To better characterize the firework-related PM_2.5_ variability, hourly average PM_2.5_ concentration data from the “during” and “after” firework periods were normalized relative to measurements from the “before” firework period. This approach borrows from Yadav et al. (2019) [16]. Based on the normalized temporal variation, we classified the counties into three categories that consisted of low, moderate, and high impact, indicating the hourly PM_2.5_ concentrations during and after the firework displays that peaked at between 1.5 and 3 times, between 3 and 5 times, and more than 5 times the level of the “before” period, respectively.

Further, with the rationale to evaluate the longer-term effect of 4th of July firework emissions on daily PM_2.5_ trends, the daily average PM_2.5_ concentrations recorded by individual sensors from June 15th to July 15th were grouped by county in both 2019 and 2020. Counties were listed in order based on the maximum 4th and 5th of July daily average PM_2.5_ levels, along with their concentrations during the control days (June 15th–July 3rd and July 6th–July 15th).

Even though the PurpleAir sensor network has a substantially larger number of samplers than the EPA’s routine PM_2.5_ monitoring network (69 monitoring sites using the Federal Equivalent Method) in California [37], the sensors still covered a small percentage of the total census tracts (e.g., 6.5% in Los Angeles in July 2019) when considering those sensors that were in operation in both 2019 and 2020. Therefore, we grouped the census tracts according to whether they were located in the northern or southern regions of California. This approach increased our statistical sample size per sub-region, which allowed us to more robustly investigate the burden of 4th of July-related PM_2.5_ and the relative impacts incurred by DACs and non-DACs as well as by urban and rural areas in different regions (i.e., southern and northern California). We evaluated the firework-related impacts on PM_2.5_ levels according to different demographic factors, SES, and disease rate (i.e., asthma) at the census tract level across the state in 2019. We focused on 2019 since the 2020 fireworks were mostly restricted to individual households due to the COVID-19 pandemic and widespread cancelations of municipal firework shows to prevent in-person gatherings, which meant that 2020 did not reflect the traditional patterns of firework activity. The statistical significance of the differences between the sensor-based PM_2.5_ concentrations and their percent increases on July 4th, according to various sociodemographic and pollution burden factors between census tracts were assessed by means of one-way ANOVA testing using pairwise comparisons. All the visualizations and data analyses related to the temporal variation of PM_2.5_ concentrations and the impact of fireworks on PM_2.5_ levels according to different socioeconomic factors were performed using Python 3.0.

Optimized hot spot analysis, a tool that identifies statistically significant spatial clusters of high values (hot spots) and low values (cold spots), was performed on the average daily PM_2.5_ concentrations on the 4th and 5th of July 2019 and 2020 using ArcGIS software (ESRI, Redland, CA, USA) and the Getis-Ord Gi* statistic [38,39]. Regular 4th of July firework festival/celebration locations reported by local authorities across California were also retrieved from patch.com, a local news and information platform, which serves as an announcement portal and gathers 4th of July firework news such as Google map locations, firework start times and durations, as well as potential restrictions on firework sales (e.g., age limitations and discrete sales periods) were announced across each county and across different cities. Their corresponding location coordinates were overlaid with hotspot results and projected onto a map using ArcGIS to evaluate the proximity of PM_2.5_ hotspots relative to municipally operated firework displays.

## 3. Results

### Spatially Varying Sensor Correction Factors for Outdoor PurpleAir PM_2.5_

A linear regression of uncalibrated, but quality controlled, PurpleAir measurements against AQS measurements had an R2 of 0.82 and a slope of 0.74. Compared to measurements from the 26 AQS monitors in this study, the PurpleAir data showed a site-specific R2 ranging from 0.23 to 0.90 and a site-specific slope from 0.34 to 0.94. The observed variations between the PurpleAir and AQS data were less pronounced than those reported by Bi et al. (2020) [23].

The GWR slopes of PurpleAir (β1 in Equation (1)) averaged at 0.53, with an interquartile range of 0.03. The largest slope was 0.57. After calibration, the overall systematic bias of PurpleAir decreased from 1.2 to 0.1 µg·m^−3^. The overall PurpleAir residual error was also reduced, reflected in the decreased standard deviation of the AQS and PurpleAir differences from 6.02 to 4.10 µg·m^−3^ (a 32% decrease). The calibration model had a 10-fold coefficient of variation R2 of 0.89 for the AQS and PurpleAir data, which is higher than the R2 of 0.83 for the uncalibrated data. As the environmental conditions in our study were similar to that of a study conducted by Bi et al. (2021), we relied on the results reported by Bi et al. (2021) for the generalized additive model-fitted relationships of the AQS and PurpleAir absolute differences and temperature, RH, operating time, and uptime. 

## 4. County-Level PM_2.5_ Trends

**Figure 1:**
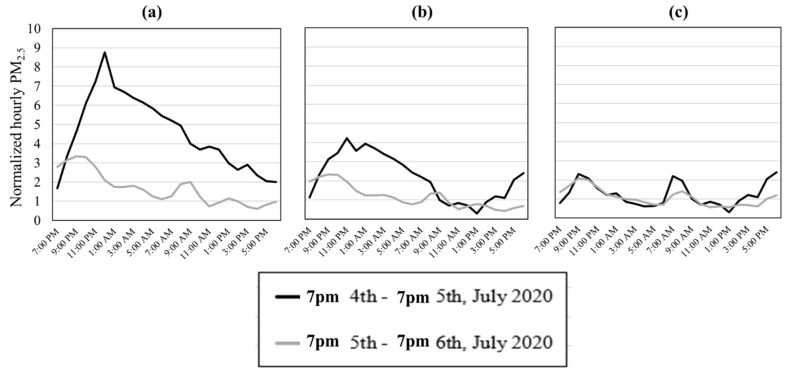
Examples of hourly average PM_2.5_ concentrations from 7:00 p.m. on July 4th to 7:00 p.m. on July 5th 2020 according to three different scenarios observed in (**a**) Los Angeles County (high impact), (**b**) Orange County (moderate impact), and (**c**) Riverside County (low impact).

Figure 1 presents examples of counties that experienced low, moderate, and high PM_2.5_ concentrations on the 4th of July, as defined by our three-tier categorization system. Figure 1a represents normalized PM_2.5_ levels for Los Angeles County in 2020, in which the PM_2.5_ concentration peaked to 5–10 times the average hourly concentration from 7:00 p.m. to midnight on July 4th, relative to the non-firework period, while Orange County in 2019 (Figure 1b) experienced a PM_2.5_ peak that was three times higher than control days on average and Riverside County in 2020 (Figure 1c) experienced only minimally elevated PM_2.5_ concentrations (hourly average peak < 1.5 times that of control days). Table S1 presents the percent of counties within different firework-related air pollution impact categories across the state. As shown, 67.4% and 81.8% of counties exhibited highly or moderately elevated PM_2.5_ concentrations during the firework period in 2019 and 2020, respectively. In addition to the total PM_2.5_ concentrations, Figure S2 shows the major peak in state-average daily filter-based trace metal concentrations (zinc, lead, and cadmium) on the 4th of July 2019, as reported by the EPA’s Air Quality System [40] known as the Chemical Speciation Network [41].

The counties with the lowest and highest peaks in daily average PM_2.5_ concentrations on July 4th and 5th 2019 and 2020 are shown in Figure S3. The results for 2020 show drastically higher peak values on the 4th of July relative to 2019. In 2019, across southern California counties, peak daily PM_2.5_ levels on the 4th of July ranged from 14.0 µg·m^−3^ in Orange County to 29.9 µg·m^−3^ in Los Angeles County. The variance was greater in 2020, as were the minimum and maximum average concentrations, ranging from 17.5 µg·m^−3^ in Ventura County to 42.6 µg·m^−3^ in Los Angeles County.

Table S2 shows county-level daily PM_2.5_ concentrations averaged over July 4th and 5th 2019 and 2020. Los Angeles County, the state’s most populous county, had the highest PM_2.5_ concentrations in both 2019 and 2020, with levels of 29.9 and 42.6 µg·m^−3^, respectively. Statewide, a moderate correlation was observed between the population density and firework related PM_2.5_ concentrations, with r = 0.45 and r = 0.56 in 2019 and 2020, respectively, across 52 counties. Further, the effect of meteorological differences between regions (northern vs. southern California), and years (2019 vs. 2020) were evaluated and minimal associations between the multiple meteorological parameters (temperature, wind speed, relative humidity, and rainfall depth) were observed. The results of comprehensive statistical analysis can be found in Table S3a,b.

### 4.1. Firework-Related PM_2.5_ Hotspots in California

**Figure 2:**
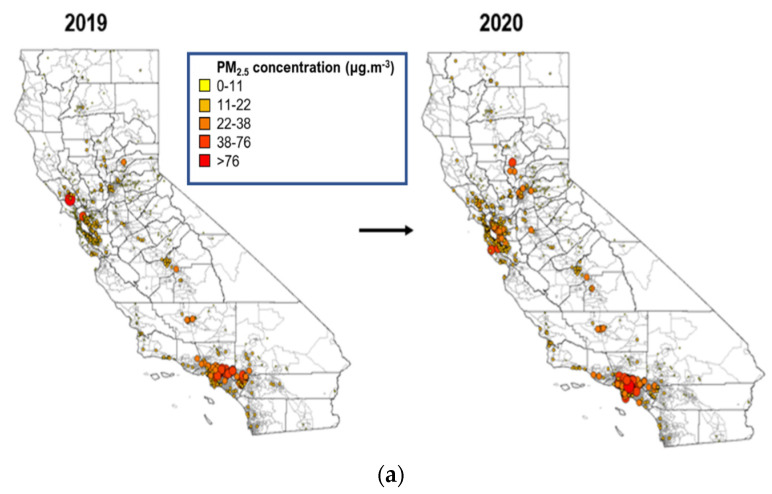

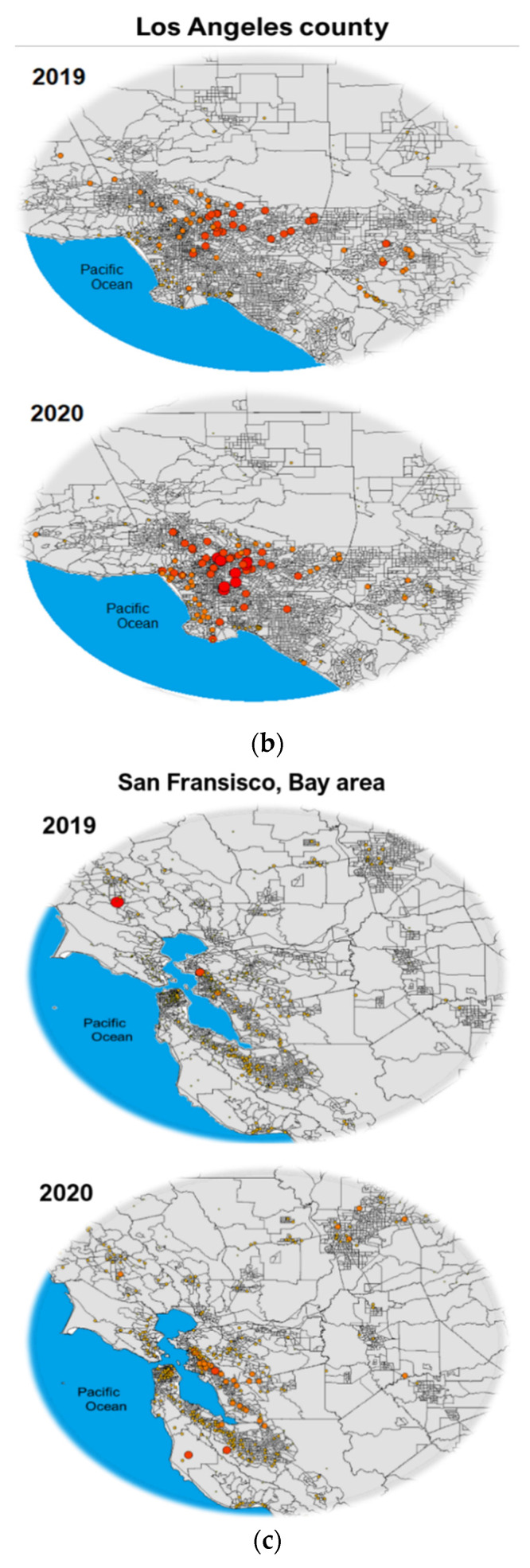

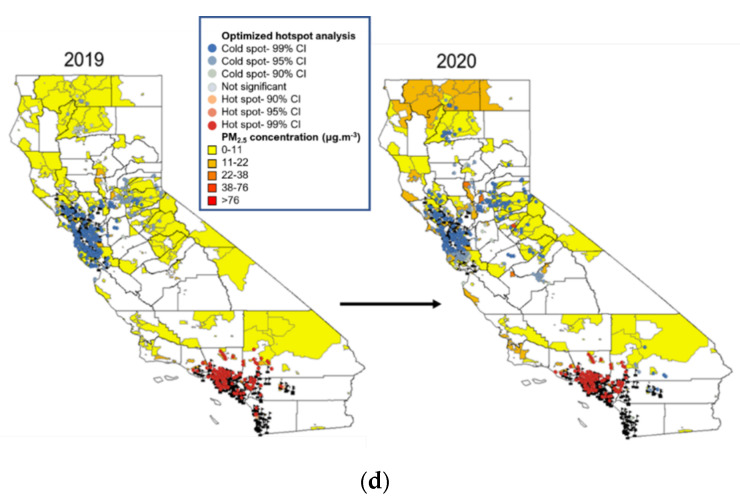
Spatial distribution of PurpleAir measured daily average PM_2.5_ concentrations across (**a**) the entire state, (**b**) Los Angeles County, and (**c**) San Francisco, Bay area scheme during the 4th and 5th July period in 2019 and 2020. Panel (**d**) shows the spatial hotspot analysis across the state for average PM_2.5_ concentration during the 4th and 5th July period in 2019 and 2020.

Figure 2a shows the spatial distribution of daily average PM_2.5_ concentrations across California on July 4th and 5th 2019 and 2020 based on all of the PurpleAir sensors that were deployed and in operation as of mid-June 2019. In general, southern California metropolitan areas experienced greater air pollution during firework episodes than northern areas such as San Francisco. Most of the sensors deployed in Los Angeles County showed daily average PM_2.5_ concentrations greater than 38 µg·m^−3^, while most other southern counties, as well as the central California area, showed concentrations below 22 µg·m^−3^ (Figure 2b). In northern California, the PM_2.5_ levels were less varied and also less elevated, with more than 80% of the counties showing concentrations between 5 and 15 µg·m^−3^, and less than 3% of sensors showing PM2.5 levels greater than 38 µg·m^−3^ near the San Francisco Bay area (Figure 2c). 

Optimized hot spot analyses based on 751 sensors in both 2019 and 2020 (Figure 2d) confirmed the observed results relating to high and low PM_2.5_ census tracts, with statistically significant (*p*-value < 0.05) hotspots in Los Angeles County, San Bernardino County and San Diego County in 2019, and a similar pattern in 2020. This is reasonable as it is also where the majority of government-operated regular firework displays were located, as shown in Figure 2d. In contrast, most of the northern California urban areas, including San Francisco County and Sacramento County, were identified as “cold spots” (*p*-value < 0.05), even in areas with designated government-operated regular firework sites. The spatial distribution of daily average PM_2.5_ concentrations using data from all of the PurpleAir sensors across California in 2020 (*n* = 2095) is shown in Figure S4. Overall, the patterns in the PM_2.5_ concentrations were similar, with results obtained using data from 751 sensors; however, additional statistically significant (*p*-value < 0.05) hotspots and high PM_2.5_ concentrations (>75 µg·m^−3^) were detected in San Luis Obispo and Santa Barbara counties during 4th and 5th of July.

### 4.2. Regulation/Restriction Effect

**Figure 3:**
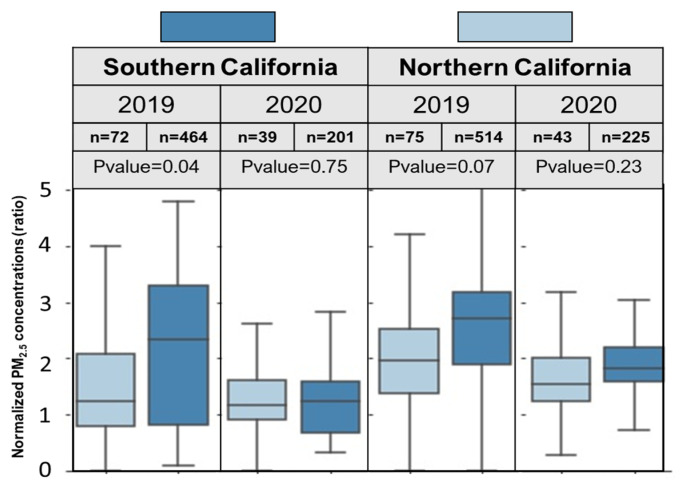
Comparison of the normalized daily PM_2.5_ concentrations (average over July 4th and 5th) in cities where firework displays were either allowed or not allowed in southern and northern California, where “*n*” shows the number of census tracts within each category and the *p*-value shows the *t*-test results between census tracts where fireworks were either allowed or not allowed in each year.

In order to quantify the effect of county- and city-wide regulations on firework-related PM_2.5_ pollution, we compared the daily average sensor data from cities where large scale firework displays were permitted to those where they were not permitted (Figure 3). In 2019, normalized PM_2.5_ levels in both northern and southern California were statistically higher (*p*-value = 0.07 and 0.04, respectively) in census tracts where fireworks were allowed, compared to those where fireworks were not allowed. In 2020, normalized PM_2.5_ levels were not statistically different between the census tracts where fireworks either were or were not allowed. 

Table 1 shows the daily PM_2.5_ concentrations averaged over July 4th and 5th 2019, and the percent increase in PM_2.5_ during this period compared to control days, according to population characteristics at the census tract level. Overall, neighborhoods with higher CES 3.0 scores had higher PM_2.5_ concentrations and higher percent increases in the daily average PM_2.5_ in comparison to control days. On average, both the PM_2.5_ concentrations and the percent increase in PM_2.5_ during the July 4th–5th period was higher in areas with higher asthma rates, higher proportions of Hispanic and African American populations, higher percentages of elderly residents (age > 65 years) and children (age < 10 years), and higher CES population characteristic scores. Mixed results were observed for poverty levels, where the census tracts with the highest amount of poverty tended to experience low and moderate PM_2.5_ impacts on the 4th of July.

In both 2019 and 2020, southern California neighborhoods experienced higher daily average PM_2.5_ levels on July 4th and 5th in comparison to northern California (Table 2), with more pronounced differences in 2020. Specifically, there was a 160.3% and 71.2% increase in two-day average PM_2.5_ concentrations during the July 4th and 5th period compared to control days in southern and northern California, respectively. Across the state and during both years, urban areas showed 1.6 and 2.1 times higher PM_2.5_ levels than those in rural areas in 2019 and 2020, respectively. Additionally, urban areas exhibited greater PM_2.5_ increases during the July 4th and 5th period (63–100% increase) compared to rural areas (35–87% increase). Similar patterns were observed between DACs and non-DACs, with DACs experiencing higher concentrations and greater increases in PM_2.5_ levels during the July 4th and 5th period; however, this difference was less pronounced than the difference between urban and rural areas. Rural communities often have lower incomes than high-income urban areas; therefore, the average urban income may not be that different to the average rural income.

## 5. Discussion

This study examined high spatiotemporal resolution PM_2.5_ concentrations due to 4th of July firework emissions in California using PurpleAir sensor network data. To our knowledge, this is one of the first studies that investigated firework-related PM_2.5_ concentrations based on large-scale sensor network measurements as well as the socioeconomic disparity in related PM_2.5_ exposures.

Our results were consistent with state-average daily trace metal concentrations and real-time hourly measurements as reported by the South Coast Air Quality Management District (SCAQMD), which showed elevated ambient concentrations of major toxic trace metals on the 4th of July 2019 based on an intensive field campaign in Compton, California [42]. In the present study, PM_2.5_ concentrations spiked dramatically during the 4th of July period, reaching levels that were ten-times above background levels in some areas. Such findings reinforce evidence that fireworks are the dominant contributors to ambient PM_2.5_ pollution during the 4th of July holiday [6,42]. After PM_2.5_ concentrations peaked on July 4th and 5th, concentrations returned to the baseline levels of mid-June and mid-July within 3–4 days in northern California and 5–6 days in southern California, which is most likely attributable to the topography of Los Angeles County, which effectively traps air pollution for extended periods. This suggests that the firework-related air pollution, while substantial, was also short term. Elevated air pollution could be even more confidently attributed to fireworks in 2020 due to the COVID-19 pandemic and related lockdown measures that resulted in lower urban emissions [43,44,45], as well as an increase in the use of household-level fireworks across the state as more people stayed at home to observe social distancing rules [46,47]. In certain counties, such as Los Angeles County, we observed over a 40% increase in daily averaged PM_2.5_ concentrations on July 4th and 5th 2020, compared to 2019. 

While population density likely plays a role in 4th-of-July-related air pollution emissions (e.g., greater fireworks abundance), it is important to note that this factor does not detract from the notion that air pollution concentrations on this day pose an environmental justice issue, since it is not necessary to reconcile the underlying causation (e.g., population density) in order to identify inequities. That is, the environmental pollution experienced disproportionately among socioeconomically disadvantaged groups, as demonstrated through spatial correlation analyses, is alone sufficient to identify issues of environmental justice, without making inferences about causation.

Spatial PM_2.5_ concentration hotspot analyses in California sub-regions showed a significant difference between northern and southern California firework-related air pollution (with concentrations in the south being higher), which may be due to the stricter regulations that prohibited municipal firework shows in most urban areas in northern California (see Table S3), as well as stricter regulations on firework sales and at-home firework use. In California, while local news reports documented a statewide increase in the use of illegal fireworks leading up to the 4th of July 2020 [39,40,41], the impact on regional PM_2.5_ levels appeared more pronounced in southern California [48,49]. On average, concentrations of PM_2.5_ in the South Coast Air Basin were 70% higher on the 4th of July 2020 than the average of previous recent years (i.e., 2017–2019) [42,43]. This finding is reinforced by SCAQMD, which reported that, despite the cancelation of public firework shows due to COVID-19, there was extensive use of personal fireworks that produced a “nonstop barrage of aerial bursts and explosions” across southern California for hours [50]. In addition to extensive use of at-home fireworks in southern California, the dry and stagnant air reported during the night of July 4th 2020 is also likely to have exacerbated the problem by trapping air pollution near the surface [42]. 

In California, while regulations vary by city and county, only non-aerial fireworks are allowed for personal use [41]. Legal consumer fireworks are also subjected to strict regulations on the selling period and purchasing age limit of such items [41]. While several highly populated counties (*n* = 15) (Table S1), such as San Diego, Contra Costa, and San Francisco, are on the list of counties where municipal firework shows are illegal [51,52], more than 80% of the counties on this list were located in northern California. Although a county may choose to legalize fireworks, in some cases cities within those counties still prohibit them [53]. For example, less than half and one-third of the cities in Los Angeles County and Orange County, respectively, allow fireworks despite them being legal at the county level. Overall, the percentage of cities with legalized fireworks was lower in northern California (~14%) compared to southern California (~29%). The lack of effective regulations in 2020, given the COVID-19 lockdown and increase in household fireworks, likely caused the observed differences between the 2019 and 2020 PM_2.5_ levels. These results underscore the importance of not only county-wide regulations but also city- and/or community-wide regulations to reduce short-term firework-related PM_2.5_ exposure. 

Firework emissions affect sub-populations and sub-regions of California differently. In general, urban census tracts with lower educational attainments, higher proportions of minority residents, and more vulnerable populations (i.e., elderly, children, and residents with respiratory disease) experienced a higher concentration of firework-related PM_2.5_ pollution, which highlights the importance of environmental justice awareness and education about the hazardous effects of short-term household-level and city-wide firework displays as well as subsequent policies and regulations related to firework permission and sales. In recent years, systematic research into the health issues caused by air pollution has been directly focused on the effects of firework-related pollutants [11,12]. Findings from studies on mental disorders have also revealed that short-term air pollution exposure is associated with an increased risk of hospitalizations related to attention-deficit/hyperactivity disorder (ADHD) [54], depression [54], schizophrenia, and epileptic seizures and/or hospitalizations [55,56,57,58,59]. Further, early research suggests that short term exposure to high air pollution concentrations could play a role in COVID-19 outcomes and exacerbations [60,61]. Given the growing number of studies suggesting a potential role of air pollution in aggravating these mental disorders, similar sub-population and sub-regional analyses need to be conducted in the context of the extremely high levels of firework-related air pollution experienced during the 4th of July [62]. 

We observed only a moderate positive correlation between poverty levels and PM_2.5_ concentrations during the 4th of July. This may be a result of poorer communities being less able to afford fireworks at both the individual and municipal level. With that said, there are likely still areas in and around poorer communities where fireworks are used, thus still contributing to air pollution and accounting for the moderate increase in PM_2.5_ levels observed in these areas during the firework season. 

Our results showed a moderate correlation between firework-related PM_2.5_ pollution and a county-level population count as well as more elevated PM_2.5_ levels in urban areas, as opposed to rural areas, implying a greater opportunity for human exposure and health effects. More importantly, in 2019, communities with the highest asthma rates saw over a 200% increase in firework-related PM_2.5_ concentrations, which has important implications for health given that short-term PM exposure can cause severe effects among asthma patients [63,64]. Higher PM_2.5_ levels were also observed in areas with greater proportions of elderly residents and children. These results highlight the potential for greater adverse effects related to firework activity and the need for increased advocacy as well as more effective firework regulations and/or enforcement in key areas. Such findings may be used to help inform the selection of lower impact municipal firework display locations and, in turn, reduce the effect of 4th-of-July-related air pollution and socioeconomic inequities related to exposure. In addition, fireworks have other widely known negative effects, such as loud explosive noise, which can trigger post-traumatic stress disorder (PTSD) in veterans [58]. The heat of combustion and explosive force of fireworks also cause injuries that yield thousands of hospitalizations each year [65,66]. Despite the short-term ambient PM_2.5_ concentrations and other adverse impacts related to fireworks, it may be challenging to fully resolve the situation given the long-held tradition of using fireworks on the 4th of July and similar holidays. For this reason, methods to reduce exposure should also include personal behavioral interventions, such as wearing proper masks during the outdoor viewing of firework displays, staying indoors with doors and windows closed, and using an air purifier in the indoor setting. Such interventions, particularly the wearing of commercial or surgical masks [67,68], would need to be paired with education to ensure efficacy (e.g., proper fitting). 

Importantly, more sustainable and eco-friendly alternatives to firework shows also exist, such as drone light shows, which have become increasingly popular in some celebration events in China [69]. Drone light shows differ from fireworks displays in that drones are reusable and do not emit either chemical or noise pollution. However, limited companies currently have the proper permits, training, technical skills, and supplies to plan and carry out large-scale drone light shows [70,71]. Government-scale investments could potentially expedite the adoption and use of such technology and, in turn, help solve the health and environmental concerns related to conventional fireworks moving forward.

This study has several strengths. First, the utilization of the dense PurpleAir network allowed for a more spatially resolved characterization of ambient PM_2.5_ concentrations that improved the ability to identify air pollution hotspots at the census tract level. This is in contrast to the majority of recent studies evaluating the effect of firework emissions on PM_2.5_ concentrations and composition, which used either readily available 24 h (filter-based) samples (Chemical Speciation Network stations [33]) or scarcely distributed samples from continuous monitoring stations (Federal Equivalent Method instrument stations [34]). Further, our study is one of the first to evaluate the effects of county- and city-wide firework regulations on ambient PM_2.5_ concentrations. Additionally, to help inform the effectiveness of future policies related to firework emissions and, in turn, optimize the benefits to public health and the environment, we evaluated the spatiotemporal variation of PM_2.5_ concentrations at the census tract level according to demographic and socioeconomic factors, disease burden, and urban vs. rural regions.

Limitations of this study include the potential for temporal mismatch between the demographic factors and the 4th of July PM_2.5_ concentration data, given that most of the population and pollution indicators from the CES 3.0 dataset were obtained before 2016, while the PurpleAir data were collected in 2019 and 2020. Additionally, PM_2.5_ levels within the census tracts were estimated using averaging measurements from all of the sensors within those tracts. Thus, the number of sensors may affect the average values, with higher uncertainties in the census tracts with a lower number of sensors. While the temporal and spatial resolution of measurements is improved significantly relative to prior studies, less than 10% of all the census tracts in California were covered by the PurpleAir sensor network as of 2019. Thus, many census tracts were not represented in this study. Additionally, given that the PurpleAir network does not represent random sampling, census tract-level analyses contain an inherent sampling bias, which limited our ability to determine finer scale variability between certain areas depending on socioeconomic factors and firework-related PM_2.5_ levels. Lastly, this study only characterized air pollution in the form of PM_2.5_ as opposed to other air pollutants, such as black carbon and gaseous pollutants.

## 6. Conclusions

This study revealed peaks and hotspots in sensor-based PM_2.5_ concentrations during the 4th of July episode at a fine temporal and spatial resolution across California. Census tract-based analyses indicated that firework-related PM_2.5_ levels varied substantially and were disproportionately elevated in communities characterized by higher proportions of minority group populations, children and elderly residents, and asthma rates, thus underscoring the importance of environmental justice and education about the hazardous effects of short-term household and city-permitted firework displays. Analyses of regional and local firework regulations across the state showcased the effectiveness of county-wide and city-wide policy in reducing short-term air pollution during the 4th of July period. Further research on socioeconomic factors among neighborhoods near regular firework shows should be carried out to help inform policies that more equitably protect the health and environment of vulnerable populations.

## Figures and Tables

**Table 1:** Description of the firework-related PM_2.5_ concentrations in 2019 according to various demographic, socioeconomic, and disease rate factors.

Characteristics	% Increase in PM_2.5_ on July 4th & 5th Relative to Control Days			PM_2.5_ Averaged over July 4th & 5th (µg·m^−3^)		
	0–100	100–200	≥200	0–22	22–38	38≥
(*n* = 673 total census tracts)	(*n* = 185)	(*n* = 234)	(*n* = 254)	(*n* = 145)	(*n* = 283)	(*n* = 245)
CES overall Score, 0–100 *	15.8	17.6	20.1	13.2	18.4	19.2
Population characteristics in CES 3.0, mean						
^1^ Asthma rate **	21.0	27.1	32.1	20.1	38.4	49.3
^2^ Educational Attainment, % **	14.3	13.2	10.2	17.8	16.2	17.2
^3^ Poverty, %	26.1	29.1	25.1	33.1	24.1	25
^4^ CES Population Characteristics Score, 0.1–10	3.3	4.1	4.8	3.1	3.6	3.9
Race/ethnicity, %						
Hispanic **	10.3	19.2	22.1	13.4	15.5	24.1
White	49.2	56.2	51.2	50.2	52.3	53.2
African American **	2.1	4.1	4.3	3.4	3.6	3.8
Native American	0.4	0.5	0.5	0.5	0.6	0.7
Asian American	13.4	14.5	16.3	14.7	13.1	13
^5^ Portion of total population	29.8	50.1	20.1	39.6	20.2	41.2
Demographic, %						
Elderly > 65 (%) *	14.3	15.4	15.8	14.1	14.5	15.4
Children < 10 (%) *	11.2	11.8	13.1	11.1	11.6	12.7

* Statistical significance of between-group differences in the results with a *p*-value < 0.1. ** Statistical significance of between-group differences in the results with a *p*-value < 0.05. ^1^ Asthma rate: an age-adjusted rate of emergency department visits for asthma. ^2^ Educational Attainment: percent of the population over the age of 25 with less than a high school education. ^3^ Poverty: percent of the population living below two times the federal poverty level. ^4^ Population characteristics score: the value is derived for each census tract using the average percentiles for three vulnerable population indicators (asthma, cardiovascular disease, and low birth weight) and five socioeconomic indicators (educational attainment, housing burdened low-income households, linguistic isolation, poverty, and unemployment). The calculated average percentile is divided by 10 for a population characteristic score ranging from 0.1–10. ^5^ Percentage of total population: percentage of the total population of 673 census tracts across the state from which PM_2.5_ concentration data was used for this analysis.

**Table 2:** Variation in PM_2.5_ concentrations before and after firework episodes at different spatial scales, urban vs. rural areas, and disadvantaged vs. non-disadvantaged communities.

Year		2019		2020	
		July 4th and 5th PM_2.5_	PM_2.5_ on July 4th Relative to Control Days (% Increase)	July 4th and 5th PM_2.5_	PM_2.5_ on July 4th Relative to Control Days (% Increase)
Northern California		15.4	56.4	19.2	71.2
	Landuse				
	Urban	22.3	95.4	26.5	130.2
	Rural	12.3	32.1	15.3	34.2
	Disadvantaged Community				
	Yes	25.7	67.4	28.3	83.2
	No	11.2	43.2	16.7	63.4
Southern California		18.4	87.2	26.2	160.3
	Landuse				
	Urban	25.4	130.4	34.5	180.2
	Rural	15.3	35.1	17.2	80.2
	Disadvantaged Community				
	Yes	27.2	95.1	31.2	172.4
	No	17.3	64.2	21.3	130.2

## Data Availability

The data presented in this study are available on request from the corresponding author.

## Supplementary Materials

The following are available online at https://www.mdpi.com/article/10.3390/ijerph18115735/s1, Table S1: Summary of the percent of counties with clear, some and no immediate impact of July 4th firework emissions on PM_2.5_ concentrations. The potential associations for each county were examined between patterns of hourly average PM_2.5_ peaks and the impact of firework display events using normalized concentrations based on hourly levels before the fireworks displays (i.e., 7:00 p.m. 3rd July–7:00 p.m. 4th July. Table S2: County-wide mean PM_2.5_ concentrations on July 4–5th across the state in (a) 2019 and (b) 2020. Counties are sorted based on high-to-low mean PM_2.5_ concentrations. The rank column shows the ranking of the county in terms of population across the state. Percentage increases higher than 100 are highlighted in both tables. Cells with “NA” indicate that there were no sensors available in these counties. Table S3: List of counties in which fireworks are prohibited county-wide. Table S4: (a) Single factor ANOVA T-test result based on city-based meteorology data (temperature, wind speed, relative humidity, and rainfall depth) for both 2019 and 2020 from National Oceanic and Atmospheric Administration (NOAA). (b) Pearson correlation coefficients between PM_2.5_ and each of the meteorological factors across the state in non-control days. Figure S1: Outdoor operational PurpleAir sensor distribution from mid-June to mid-July 2019. Figure S2: State-average daily filter-based selected trace metal concentrations during 4th July firework episode in 2019. Data includes average selected trace metal (more than 75% of data during July 1–August 1 2018 and 2019) concentration from 10 operating sites (out of 26 sites) in Chemical Speciation Network (CSN) across California during June–August 2018–2019. Figure S3: Temporal variation of summary PM_2.5_ concentrations across counties with the lowest, highest, and median levels during the 4th of July firework episode in both (a) 2019 and (b) 2020. Red and blue lines indicate the temporal variation in daily PM_2.5_ concentrations for the counties with the highest and lowest 4–5th July average PM_2.5_ concentrations, respectively. The dashed line in each figure shows the median of the daily PM_2.5_ levels across the 52 counties. Figure S4: (a) Spatial distribution of daily average PM_2.5_ concentration on July 4–5th and (b) hotspot areas across California in 2020 based on the PurpleAir sensors that were in operation since June 2019 versus all the active ones since June 2020. Figure S5: Average temporal variation of hourly PM_2.5_ elevation based on non-control days hourly averages for near to regular firework location (near source), near freeway and the remaining sensors.
