# Supplementary material for: Impact of 4th of July Fireworks on Spatiotemporal PM2.5 Concentrations in California Based on the PurpleAir Sensor Network: Implications for Policy and Environmental Justice
Source: Int J Environ Res Public Health. 2021 May 27;18(11):5735. doi: 10.3390/ijerph18115735 (PMC8198140; doi:10.3390/ijerph18115735)
Supplement: Supplementary file 1 [file ijerph-18-05735-s001.zip › ijerph-1160767-supplementary.pdf]

Impact of 4th of July fireworks on spatiotemporal $PM_{2.5}$ concentrations in California based on the PurpleAir sensor network: Implications for Policy and Environmental Justice

Table S1. Summary of the percent of counties with clear, some and no immediate impact of July 4th firework emissions on $PM_{2.5}$ concentrations. Potential associations for each county were examined between patterns of hourly average $PM_{2.5}$ peaks and impact from firework display events using normalized concentration based on hourly levels before fireworks displays (i.e., 7pm 3rd July -7pm 4th July).

Year	Clear impact	Some impact	No immediate impact
2019	31.2%	36.2%	32.6%
2020	57.3%	24.5%	18.2%

Table S2. County-wide mean $PM_{2.5}$ concentrations on July4-5th across the state in a) 2019 and b) 2020. Counties are sorted based on high-to-low mean $PM_{2.5}$ concentration. Rank column shows the ranking of the county in terms of population across the state. Percentage increase more than 100 are highlighted in both tables. Cells with "NA" indicate that there were no sensors available in these counties.

a)

County	Control days average	4-5th July average	Percentage Increase (%) to baseline	County Rank in population
Los Angeles	19.1	29.9	56.4	1
Riverside	18.4	26.9	46.5	4
Ventura	13.2	16.9	28.3	13
Orange	12.2	14.1	15.2	3
San Diego	11.5	11.7	1.4	2
Santa Barbara	9.4	12.0	27.8	20
Tulare	8.6	15.0	74.4	18
Sonoma	8.6	15.9	84.3	17
Imperial	7.9	7.6	-4.8	30
Kern	7.3	16.9	132.7	11
Alameda	7.1	12.7	79.6	7
San Francisco	7.0	9.0	27.3	12
San Bernardino	6.8	11.0	61.3	5
Nevada	6.4	9.4	48.1	36
San Luis Obispo	6.3	8.2	29.9	23
Fresno	6.2	14.7	136.1	10
Contra Costa	5.9	10.4	75.9	9
Del Norte	5.9	8.2	39.8	49
Santa Clara	5.6	11.0	96.1	6
San Benito	5.6	15.4	174.7	42
Napa	5.5	9.1	64.5	34
Monterey	5.5	9.2	65.9	21
Solano	5.4	8.7	62.8	19
Glenn	5.3	13.9	161.7	48

Sutter	5.3	15.1	186.2	37
Mariposa	5.2	8.2	55.9	53
Santa Cruz	5.1	8.6	68.3	25
Madera	5.1	7.3	44.3	32
San Mateo	5.0	7.8	55.3	14
Butte	5.0	9.7	94.2	28
Sacramento	4.8	10.6	121.2	8
Yuba	4.8	10.5	120.5	39
Stanislaus	4.8	13.1	176.7	16
Yolo	4.7	10.6	126.8	27
Marin	4.6	6.4	38.2	26
Placer	4.4	9.4	110.5	22
Humboldt	4.4	5.3	21.1	35
Tehama	4.3	10.2	137.4	40
Merced	4.3	9.3	116.2	24
Amador	4.0	7.8	93.5	46
Tuolumne	4.0	6.8	70.5	43
Shasta	3.9	6.0	52.5	31
San Joaquin	3.9	9.6	143.3	15
El Dorado	3.9	7.0	81.1	29
Mendocino	3.8	5.7	48.6	38
Calaveras	3.8	6.5	74.1	44
Plumas	3.7	6.8	82.0	51
Trinity	3.5	7.1	106.8	55
Modoc	3.3	5.8	74.5	56
Lake	3.3	5.9	79.2	41
Mono	3.2	3.8	17.8	54
Siskiyou	3.1	5.6	79.1	45
Lassen	2.8	5.2	84.3	47
Inyo	2.7	3.3	18.9	52
Alpine	2.2	5.1	129.2	58
Sierra	NA	NA	NA	57
Colusa	NA	NA	NA	50
Kings	NA	NA	NA	33

b)

County	Control days average	4-5th July average	Percentage Increase (%) to baseline	County Rank in population
San Luis Obispo	22.6	29.7	31.5	23
Los Angeles	14.3	42.6	197.3	1

Calaveras	13.5	15.5	15.4	44
Riverside	13.4	15.4	15.0	4
Ventura	11.7	17.5	50.4	13
San Diego	10.7	13.9	29.6	2
Orange	10.1	17.3	71.3	3
Santa Clara	9.9	18.2	82.8	6
Imperial	9.3	4.7	-49.6	30
Tulare	8.8	17.2	94.9	18
Santa Barbara	8.7	18.8	116.3	20
Del Norte	7.8	15.9	104.8	49
Tehama	7.1	14.0	97.2	40
San Francisco	6.9	15.8	128.0	12
Kern	6.6	17.9	170.0	11
San Bernardino	6.5	7.7	18.0	5
Contra Costa	6.5	16.1	148.1	9
Fresno	6.5	13.9	114.3	10
Mariposa	6.4	6.9	6.8	53
Alameda	6.3	21.3	240.3	7
Monterey	5.6	12.1	117.0	21
San Mateo	5.5	14.9	169.1	14
Sonoma	5.2	14.3	172.8	17
Madera	5.1	5.8	12.9	32
Sutter	5.1	20.9	307.3	37
San Benito	5.1	19.9	288.2	42
Santa Cruz	4.9	12.1	147.3	25
Napa	4.8	12.3	158.6	34
Sacramento	4.7	17.9	277.7	8
Yuba	4.7	12.2	160.0	39
Merced	4.7	12.5	166.6	24
Butte	4.7	10.0	113.2	28
Glenn	4.7	12.1	159.7	48
Solano	4.6	14.3	207.6	19
Humboldt	4.5	8.5	88.5	35
Yolo	4.3	14.2	228.6	27
Marin	4.2	11.7	176.7	26
Stanislaus	4.2	17.3	315.1	16
Mendocino	4.0	11.0	178.9	38
San Joaquin	3.9	13.9	253.0	15
Placer	3.9	9.5	141.6	22
El Dorado	3.7	5.4	44.7	29
Plumas	3.7	4.3	16.6	51
Nevada	3.6	3.4	-5.2	36

Amador	3.6	9.4	161.5	46
Tuolumne	3.4	4.9	43.3	43
Inyo	3.3	3.1	-5.5	52
Mono	3.1	1.8	-44.0	54
Shasta	3.0	7.4	146.6	31
Lake	2.9	7.3	153.9	41
Modoc	2.8	8.9	221.1	56
Trinity	2.6	9.6	275.5	55
Siskiyou	2.5	11.9	371.7	45
Lassen	2.3	3.3	44.7	47
Alpine	2.2	1.4	-36.2	58
Sierra	NA	NA	NA	57
Colusa	NA	NA	NA	50
Kings	NA	NA	NA	33

Table S3. List of counties in which fireworks are prohibited county-wide.

**County name**	**Population/mile^2**	**Region**
San Francisco	17,179	Northern California
Contra Costa	1,465	Northern California
San Diego	736	Southern California
Marin	485	Northern California
Napa	182	Northern California
El Dorado	106	Northern California
Nevada	103	Northern California
Shasta	47	Northern California
Mendocino	25	Northern California
Tuolumne	25	Central California
Mariposa	13	Central California
Plumas	8	Northern California
Trinity	4	Northern California
Sierra	3	Northern California
Alpine	2	Northern California

Table S4. a) Single factor ANOVA T-test result based on city-based meteorology data (temperature, wind speed, relative humidity, and rainfall depth) for both 2019 and 2020 from National Oceanic and Atmospheric Administration (NOAA). b) Pearson correlation coefficients between $PM_{2.5}$ and each of the meteorological factors across the state in non-control days

a)

Control vs on-control	Temprture (F)		Relative Humidity (%)		Wind Speed (m/s)		Rain Depth (inch)	
Groups	**No-control**	**Control**	**No-control**	**Control**	**No-control**	**Control**	**No-control**	**Control**
Count	492	492	492	492	492	492	492	492
Average	74	72	52	57	0.08	0.07	0.02	0.05
Variance	24	30	14	17	0.03	0.04	0.01	0.03
P-value	0.12		0.23		0.35		0.78	
**Regional during non-control days**								
Groups	**NorthCal**	**SouthCal**	**NorthCal**	**SouthCal**	**NorthCal**	**SouthCal**	**NorthCal**	**SouthCal**
Count	278	214	278	214	278	214	278	214
Average	79	81	62	58	0.08	0.07	0.06	0.03
Variance	26	35	12	10	0.03	0.02	0.01	0.01
P-value	0.09		0.21		0.23		0.12	
**Yearly during no-control days**								
Groups	**2019**	**2020**	**2019**	**2020**	**2019**	**2020**	**2019**	**2020**
Count	492	492	492	492	492	492	492	492
Average	73	74	49	52	0.07	0.08	0.03	0.02
Variance	27	24	10	14	0.03	0.03	0.02	0.01
P-value	0.23		0.35		0.17		0.56	

b)

	PM2.5	Temprature	Wind speed	Rainfall Depth	Relative Humidity
PM2.5	1				
Temprature	**0.34**	1			
Wind speed	-0.23	0.03	1		
Rainfall Depth	-0.36	-0.41	-0.12	1	
Relative Humidity	0.12	-0.23	0	**0.57**	1

Figure S1. Outdoor operational PurpleAir sensor distribution in mid-June to mid-July 2019.

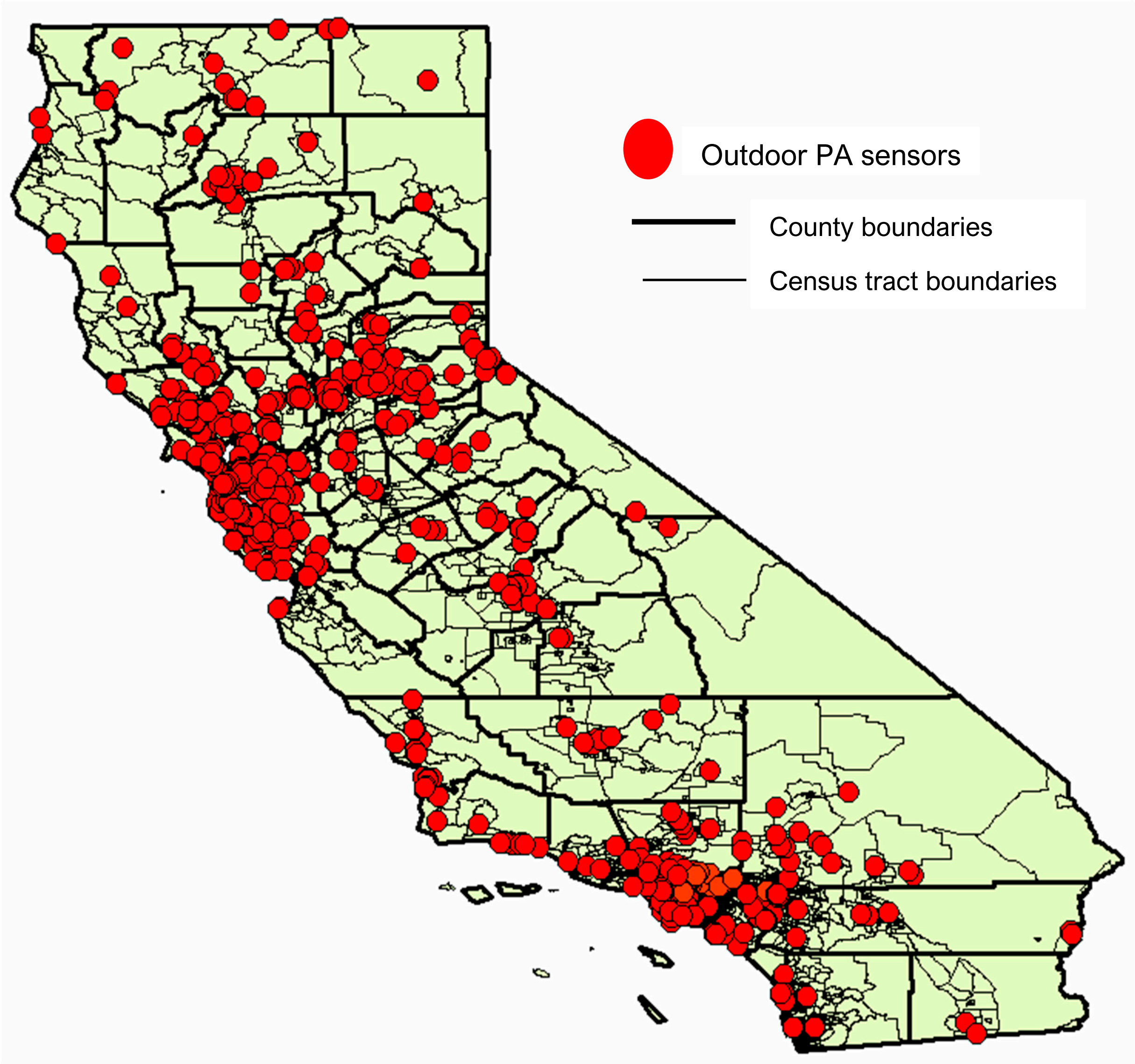

Figure S2. State-average daily filter-based selected trace metal concentrations during 4th July firework episode in 2019. Data includes average selected trace metal (more than 75% of data during July 1-August 1 2018 and 2019) concentration from 10 operating sites (out of 26 sites) in Chemical Speciation Network (CSN) across California during June-August 2018-2019.

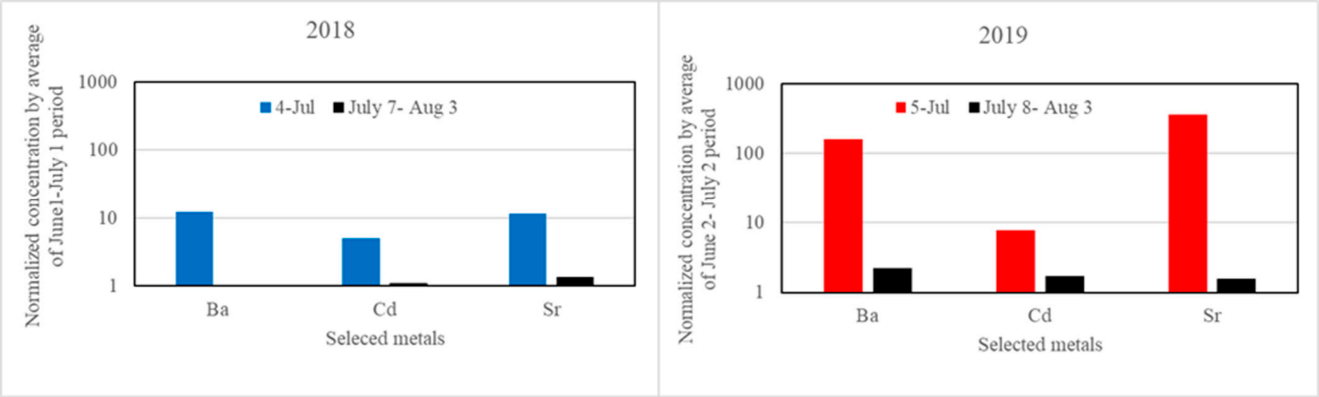

Figure S3. Temporal variation of summary $PM_{2.5}$ concentrations across counties with lowest, highest and median during 4th of July firework episode in both a) 2019 and b) 2020. Red and blue lines indicate temporal variation of daily $PM_{2.5}$ for the counties with highest and lowest 4-5th July average $PM_{2.5}$ concentration, respectively. Dashed line in each figure shows the median of the daily $PM_{2.5}$ levels across the 52 counties.

a)

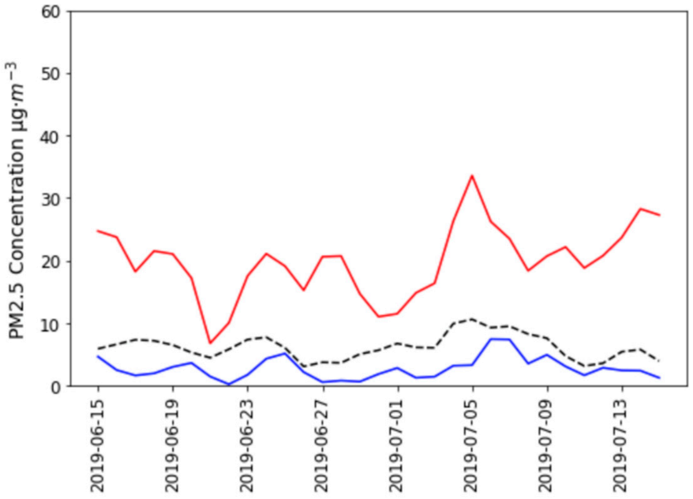

b)

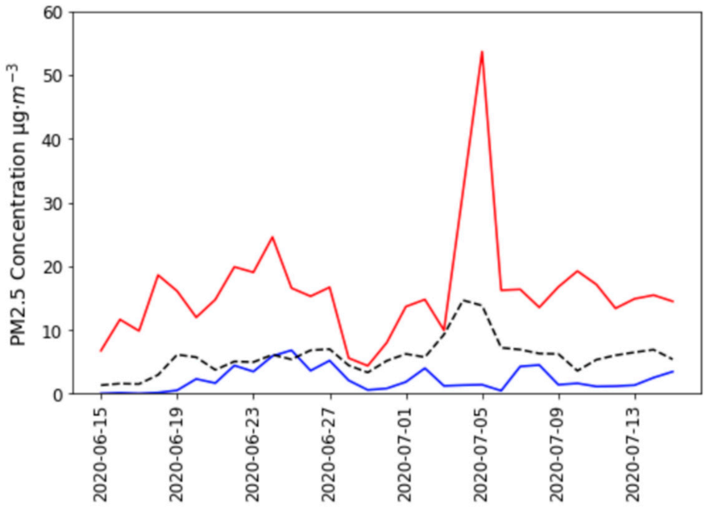

Figure S4. a) Spatial distribution of daily average $PM_{2.5}$ concentration on July 4-5th and b) hotspot areas across California in 2020 based on PurpleAir sensors that were in operation since June 2019 *versus* all the active ones since June 2020.

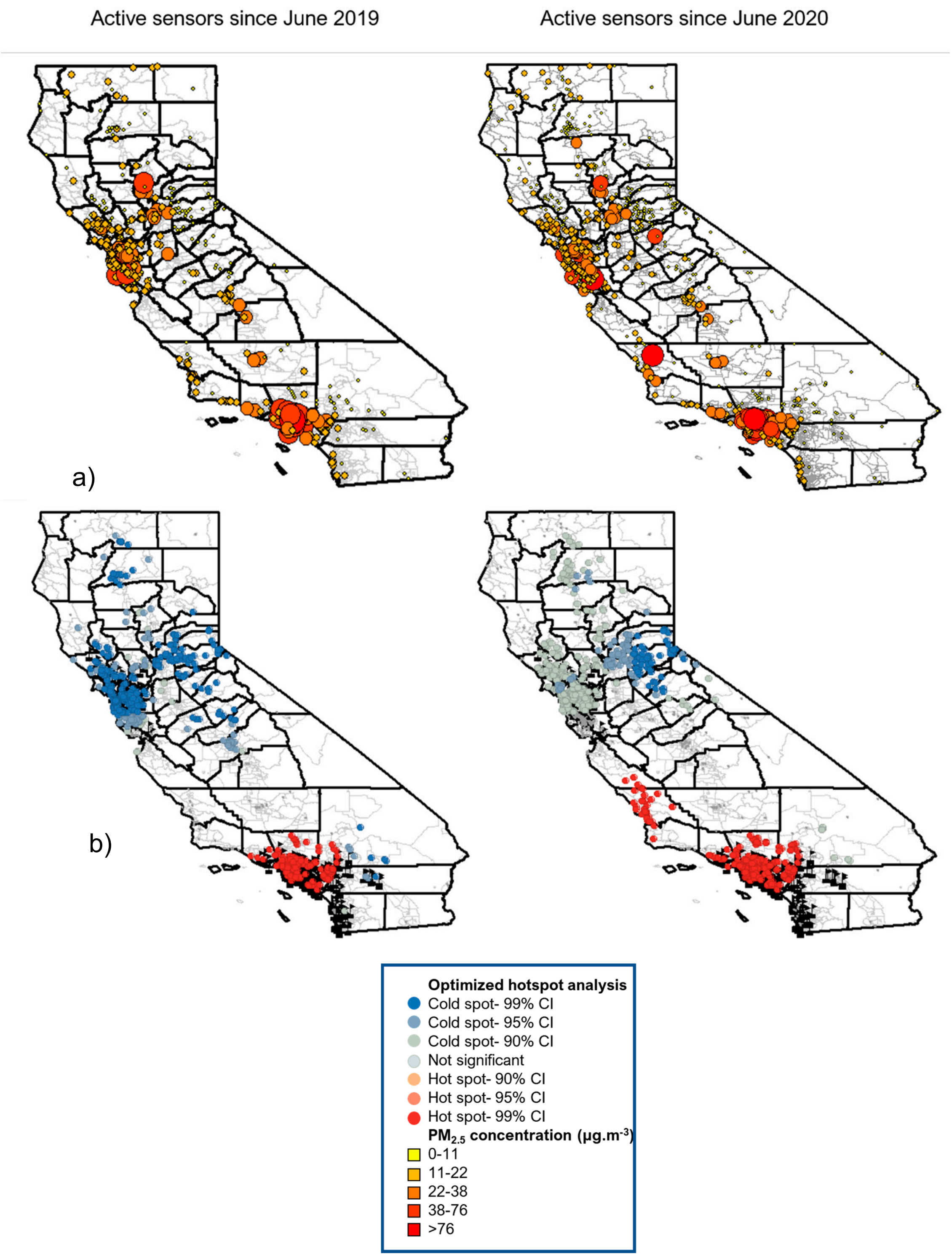

Figure S5. Average temporal variation of hourly $PM_{2.5}$ elevation based on non-control days hourly averages for near to regular firework location (near source), near freeway and the remaining sensors.

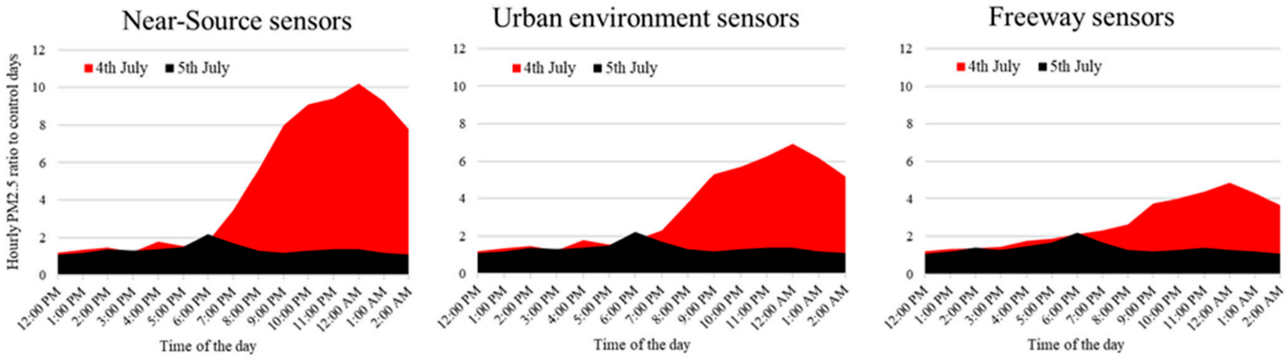
Near-Source sensors
Urban environment sensors
Freeway sensors
4th July
5th July
Hourly PM2.5 ratio to control days
Time of the day
12:00 PM
1:00 PM
2:00 PM
3:00 PM
4:00 PM
5:00 PM
6:00 PM
7:00 PM
8:00 PM
9:00 PM
10:00 PM
11:00 PM
12:00 AM
1:00 AM
2:00 AM
